# The double-edged sword of SASP in breast cancer: from tumor suppression to progression and therapy resistance

**DOI:** 10.3389/fonc.2026.1853883

**Published:** 2026-08-25

**Authors:** Xianneng Gao, Yunlong Zhang, Yue Sun, Tiefeng Xu

**Affiliations:** Department of Breast Surgery, Hainan Affiliated Hospital of Hainan Medical University (Hainan General Hospital), Haikou, China

**Keywords:** BC, mechanisms, SASP, The double-edged sword, therapy

## Abstract

The senescence-associated secretory phenotype (SASP) represents a complex and dynamic collection of bioactive factors secreted by senescent cells, which exerts dual regulatory effects on breast cancer (BC) progression. When SASP is transiently expressed, it promotes tumor suppression by recruiting immune cells to enhance immunosurveillance against senescent cells, and by transmitting paracrine signals that inhibit cancer cell proliferation. In contrast, when senescent cells cannot be cleared in a timely manner and SASP transitions to a chronic, persistent state, it drives tumor progression through multiple mechanisms, including the promotion of chronic inflammation, induction of epithelial-to-mesenchymal transition (EMT), and facilitation of immune evasion. Unlike previous pan-cancer reviews that largely rely on descriptive literature summaries, this review focuses specifically on BC and integrates pathogenic mechanism analysis with inter-subtype comparisons across BC subtypes. The dual roles of SASP in BC are systematically examined along three dimensions: cellular origin, temporal dynamics, and SASP component heterogeneity. Furthermore, existing SASP-targeted therapeutic strategies are discussed, with the aim of providing novel insights into the potential role of SASP in BC treatment.

## 1 Introduction

Breast cancer (BC) consistently ranks as the first or second most common cancer worldwide. Despite geographic disparities in incidence, it remains a leading driver of the global cancer burden, with projections indicating that new cases and deaths will rise by 38% and 68%, respectively, by 2050 (1). Its incidence continues to rise, imposing a substantial disease burden. The pathogenesis of BC is multifactorial and complex, encompassing genetic alterations, hormonal exposure, and various forms of programmed cell death such as ferroptosis and pyroptosis, all of which have been extensively investigated (2, 3). In contrast to these well-characterized mechanisms, cellular senescence and its accompanying senescence-associated secretory phenotype (SASP) have emerged as equally important regulators of BC initiation and progression. Notably, the effects of SASP are dualistic in nature, and the underlying mechanisms as well as potential SASP-targeted therapeutic strategies remain incompletely understood and warrant further investigation.

Ungvari Z. and colleagues investigated the prognostic significance of aging-related genes in BC and revealed that cellular senescence plays an important role in BC progression and survival (4). SASP, one of the key features of cellular senescence, have been increasingly demonstrated to exert complex effects in cancer. Although numerous reviews have addressed the interplay between cellular senescence and the SASP in cancer, significant gaps remain. Most adopt a pan-cancer perspective without delving into the dual effects of the SASP or the molecular mechanisms that underpin them. This void is particularly conspicuous in breast cancer, which exhibits profound subtype heterogeneity, encompassing luminal A, HER2-positive (HER2+) BC, and triple-negative breast cancer (TNBC), with markedly divergent senescence-related gene expression profiles across subtypes. Consequently, pan-cancer findings cannot be directly extrapolated to elucidate SASP functions in this disease. Concurrently, therapeutic strategies targeting the breast cancer SASP are gaining traction, yet few reviews have systematically integrated subtype-specific mechanisms with emerging senescence-directed treatment modalities. This review aims to explore the dual role and underlying mechanisms of SASP in BC, offering new insights for its treatment.

## 2 Overview of SASP

SASP refers to a complex collection of bioactive substances secreted by senescent cells, comprising inflammatory cytokines, chemokines, proteases, and matrix remodeling factors produced by various cell types within the tumor microenvironment (TME) (**Table 1**). However, owing to the considerable heterogeneity of SASP, its composition and functional repertoire may vary substantially across different cancer types and tumor subtypes (26, 27). Notably, the expression levels of senescence-associated genes have been shown to be significantly higher in HER2+ BC and TNBC compared with those in Luminal A subtype BC (28). Furthermore, an analysis based on The Cancer Genome Atlas (TCGA) database demonstrated that TNBC can be stratified into distinct prognostic subgroups according to the expression profiles of senescence-associated genes (29). These findings underscore the importance of conducting SASP-related research within the specific context of BC subtypes, rather than extrapolating directly from pan-cancer studies. In BC, senescent cancer cells are considered to participate throughout the entire course of tumor initiation, metastasis, dormancy, and recurrence. They are widely detected in metastatic lesions across various molecular subtypes, with their distribution patterns correlating with the histological type of the primary tumor (28). SASP exhibits pleiotropic effects in cancer biology, capable of either promoting tumor development or suppressing tumor growth in a highly context-dependent manner (26). It plays a critical role in shaping the TME, and the induction of cellular senescence—along with the subsequent elaboration of SASP—can be triggered and regulated by diverse stress stimuli.

**Table 1:** Major components of SASP and their roles in BC.

Classification	Components	Cellular source	BC subtypes	Biological effect	Mechanism	Level of evidence	References
Pro-inflammatory cytokines	IL-6, IL-8	Senescent cancer cells	HR+ BC	Tumor-promoting	Promotion of population-level EMT and acquisition of stemness-like traits, increasing tumor invasiveness.	C	(5)
		Senescence-like CAFs (TSPAN8+ myCAFs)	BC (patients)	Tumor-promoting	Enhance stemness of surrounding BC cells through paracrine JAK/STAT3 signaling, contributing to drug resistance.	A	(6)
	IL-8	Senescent cancer cells	BC (palbociclib-induced)	Tumor-suppressive	Recruits and activates neutrophils at the tumor site, contributing to phagocytic clearance of senescent cancer cells.	C	(7)
	IL-6	Cancer cells (IL-6 as SASP component)	TNBC	Tumor-promoting	Suppresses MSN expression, leading to reduced STAT3 phosphorylation and maintenance of TNBC stemness.	C	(8)
	IL-1α	Tumor cells	TNBC	Tumor-promoting	Recruits MDSCs, inhibits CTLs/NKs, and upregulates chemokines.	B	(9)
	TGF-β	Senescent cells in TME	TNBC	Tumor-promoting	Synergizes with TNFα, suppresses immune cell function, and reprograms the TME.	B	(10, 11)
		Senescent breast fibroblasts	BC (primary fibroblasts)	Tumor-suppressive	Mediates paracrine transmission of senescence to adjacent neighboring cells via integrin β3/TGF-β pathway, confining local tumor expansion.	C	(12)
		CAFs	ER+ BC	Tumor-promoting	Suppresses ER-α expression via TGF-β signaling, releasing the brake on EMT, heightening malignant phenotype, and diminishing endocrine therapy responsiveness.	C	(13)
	TNFα	Senescent cancer cells	ER+ BC	Tumor-suppressive	Recruits CD4+ T cells and promotes Th1 polarization; senescent cancer cells upregulate TNFR1, rendering them susceptible to TNFα-mediated immune killing.	C	(14)
		Inflammatory cells	BC (pan-subtype)	Tumor-promoting	Synergizes with TGF-β to promote the expression of MMPs and EMT.	A	(11)
Chemokines	CCL2	CAFs	HR+ BC	Tumor-promoting	Upregulates MCT1 to promote lactate uptake by cancer cells, while recruiting monocytes to foster an inflammatory microenvironment.	C	(15)
	CCL5	BC cells	BC (pan-subtype)	Tumor-promoting	Increases the proportion of Treg cells to form an immunosuppressive microenvironment.	B	(16)
	CXCL1	BC cells (Zeb1-driven)	BC (pan-subtype)	Tumor-promoting	Induces macrophage polarization to the M2 phenotype and suppresses T cell-mediated anti-tumor immunity.	C	(17)
	CXCL10	Senescent endothelial cells/BC cells	BC (pan-subtype)	Tumor-promoting	Promotes tumor cell proliferation and angiogenesis, thereby driving tumor progression.	C	(18)
Growth factors	VEGF	Senescent cells (normal + cancer)	BC (pan-subtype)	Tumor-promoting	Stimulates neoangiogenesis within the tumor, creating favorable conditions for BC metastasis.	D	(19)
	FGF	BC cells	HR+ BC	Tumor-promoting	Participates in the proliferation of BC cells.	C	(20)
	PDGF	BC cells	TNBC	Tumor-promoting	By activating the NF-κB pathway to upregulate VEGF and MMP-9, it promotes tumor cell proliferation, inhibits apoptosis, and enhances migration and invasion abilities.	C	(21)
	GM-CSF	BC cells	HR+ BC	Tumor-promoting	In the acidic TME, it depletes L-arginine, which is essential for T cells, via the JAK/STAT3 and p38 MAPK pathways, thereby suppressing anti-tumor immunity and promoting tumor progression.	B	(22)
	GM-CSF, bFGF	Senescent fibroblasts	BC (pan-subtype)	Tumor-promoting	Activate the JNK signaling pathway in BC cells, inducing EMT and significantly enhancing migratory capacity and distant metastasis.	C	(23)
Matrix remodeling enzymes	MMPs	Senescent cells in TME	BC (pan-subtype)	Tumor-promoting	Degrades the ECM and promotes invasion.	A	(11)
Others	SAA	BC cells/stromal cells	TNBC	Tumor-promoting (context-dependent)	By activating the NLRP3 inflammasome, it promotes the release of pro-inflammatory factors and induces apoptosis and necrosis of tumor cells.	B	(24)
	SAA1	Senescent cancer cells	BC (palbociclib-induced)	Tumor-suppressive	Upregulated during palbociclib-induced senescence; recruits and activates neutrophils for phagocytic elimination of senescent tumor cells.	C	(7)
	HMGB1	Senescent cells/tumor cells	BC (pan-subtype)	Tumor-promoting	Promotes M2 macrophage polarization, establishes an immunosuppressive microenvironment, and facilitates tumor progression.	B	(25)

IL-6, Interleukin-6; IL-8, Interleukin-8; IL-1α, Interleukin-1alpha; TGF-β, Transforming Growth Factor-beta; TNFα, Tumor Necrosis Factor-α; CCL2, Chemokine (C-C motif) ligand 2; CCL5, Chemokine (C-C motif) ligand 5; CXCL1, Chemokine (C-X-C motif) ligand 1; CXCL10, Chemokine (C-X-C motif) ligand 10; VEGF,Vascular Endothelial Growth Factor; FGF, Fibroblast Growth Factor; BC, breast cancer; PDGF, Platelet-Derived Growth Factor; GM-CSF, Granulocyte-Macrophage Colony-Stimulating Factor; TME, tumor microenvironment; MMPs, Matrix Metalloproteinases; ECM, extracellular matrix; SAA, Serum Amyloid A; HMGB1, High Mobility Group Box.

## 3 Factors influencing the dual roles of SASP in BC

**Figure 1:**
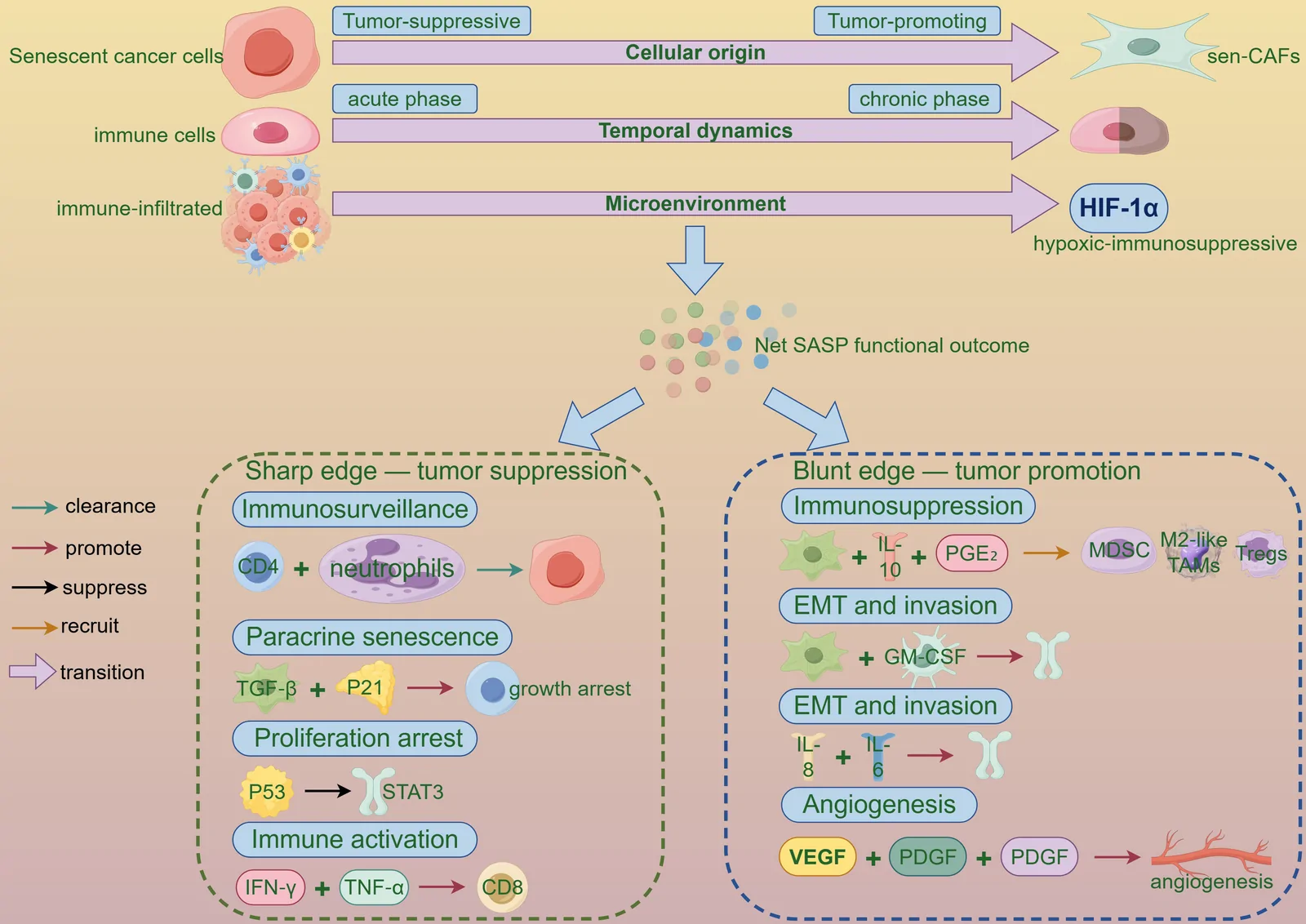
Functional integration of the SASP in breast cancer. The net functional outcome of the SASP is determined by the convergence of three axes spanning a tumor-suppressive-to-tumor-promoting continuum: the cell-of-origin axis from senescent cancer cells to sen-CAFs, the temporal axis from acute immunostimulation to chronic immunosuppression, and the microenvironmental axis from immune infiltration to hypoxic immunosuppression. When all three axes favor tumor suppression, the SASP exerts antitumor effects through immune surveillance mediated by CD4^+^ T cells and neutrophils that clear senescent cells, paracrine senescence induced by TGF-β via p21-mediated growth arrest, proliferation inhibition through p53/p21 activation and JAK2/STAT3 suppression, and immune activation via IFN-γ and TNF-α that enhance CD8^+^ T cell cytotoxicity. Conversely, when all three axes favor tumor promotion, the SASP drives progression through immunosuppression mediated by IL-10, TGF-β, and PGE_2_ that recruit MDSCs, M2-like TAMs, and Tregs, EMT and invasion via TGF-β and GM-CSF-activated JNK signaling, cancer stem cell property acquisition through IL-6 and IL-8-activated JAK/STAT3, and angiogenesis promoted by VEGF, PDGF, and CXCL10. SASP, senescence associated secretory phenotype; sen-CAFs, senescence-associated cancer-associated fibroblasts; TGF-β, transforming growth factor-β; p21, cyclin-dependent kinase inhibitor 1A; p53, tumor protein p53; JAK2, Janus kinase 2; STAT3, signal transducer and activator of transcription 3; IFN-γ, interferon-γ; TNF-α, tumor necrosis factor-α; IL-10, interleukin-10; PGE_2_, prostaglandin E_2_; MDSCs, myeloid-derived suppressor cells; TAMs, tumor-associated macrophages; Tregs, regulatory T cells; EMT, epithelial–mesenchymal transition; GM-CSF, granulocyte-macrophage colony-stimulating factor; JNK, c-Jun N-terminal kinase; IL-6, interleukin-6; IL-8, interleukin-8; VEGF, vascular endothelial growth factor; PDGF, platelet-derived growth factor; CXCL10, C-X-C motif chemokine ligand 10.

We have clearly recognized that SASP exerts dual effects in BC. Under what circumstances does SASP promote or suppress tumor progression, respectively? The functional outcome of SASP is critically dependent on the biological context in which cellular senescence occurs. First, the cellular origin of senescent cells constitutes a major determinant of SASP function. Senescence of BC cells themselves represents an intrinsically tumor-suppressive state, which further elicits antitumor immunosurveillance to amplify the suppressive effect. In contrast, senescence within the cancer-associated fibroblast (CAF) compartment predominantly generates tumor-promoting SASP factors that facilitate cancer progression (30). Second, SASP is a dynamically evolving secretome whose composition shifts temporally. During the early phase of senescence, SASP components are primarily immunostimulatory and promote immune surveillance, whereas in the late phase, they transition toward an immunosuppressive profile that facilitates immune evasion (31, 32). This temporal heterogeneity of SASP carries important therapeutic implications, suggesting the existence of a defined therapeutic window for clinical intervention. Finally, the functional outcome of SASP is not solely determined by the secretory phenotype itself but is also profoundly influenced by the tumor and its microenvironment. Within spatially distinct microenvironments, identical SASP components may exert divergent effects; for instance, SASP is more likely to exert tumor-suppressive effects in microenvironments enriched with immune cell infiltration (33). Notably, therapy-induced senescence (TIS) constitutes another principal source of pathological SASP in the clinical setting, triggered by chemotherapy, radiotherapy, endocrine therapy, and targeted agents alike. Although this response initially exerts tumor-suppressive effects, it gradually transitions to a tumor-promoting role through SASP-mediated remodeling of the tumor microenvironment (**Figure 1**).

## 4 The “sharp edge” of SASP: tumor-suppressive effects in BC

**Figure 2:**
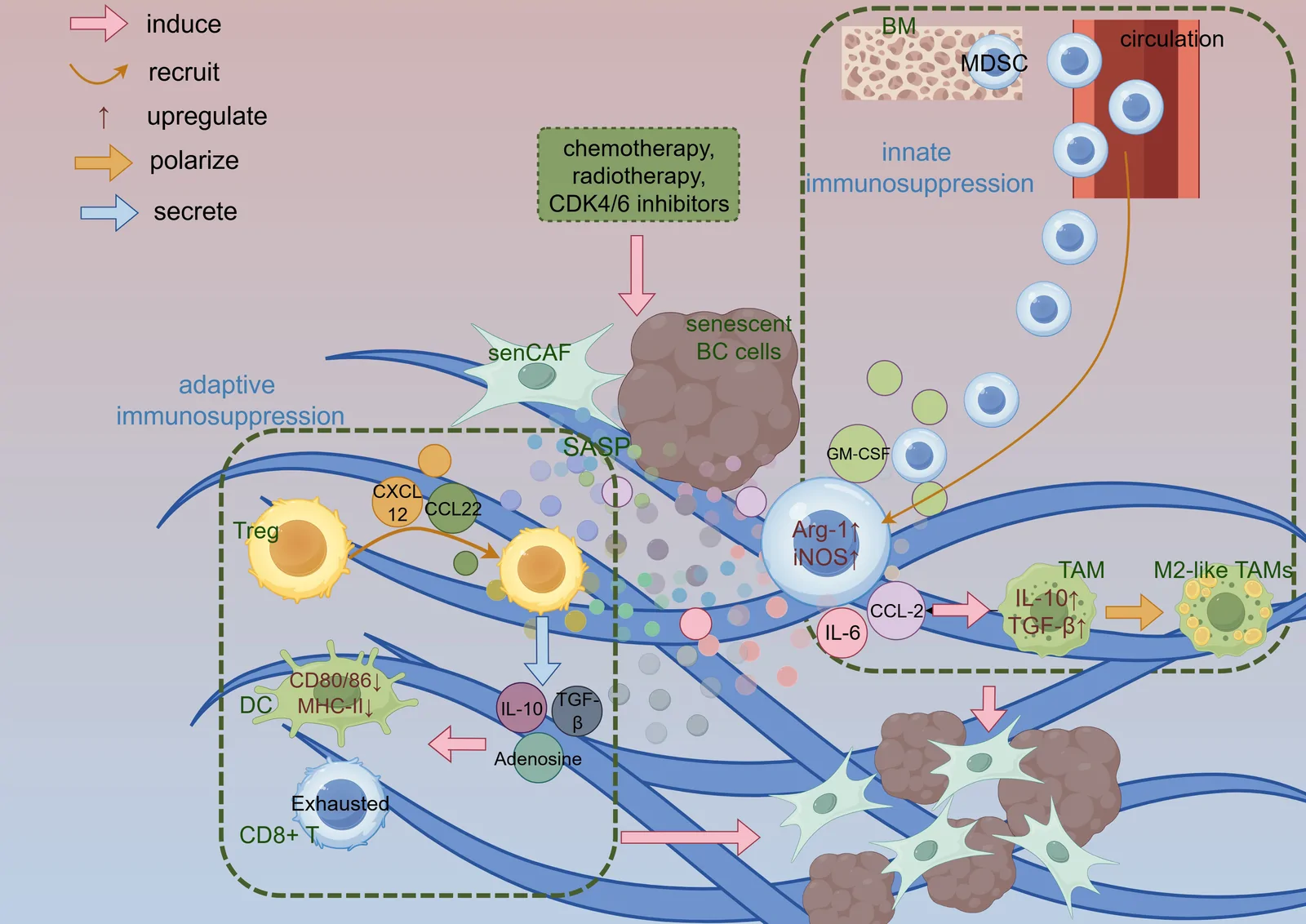
The immunosuppressive cascade mediated by SASP. Chemotherapy, radiotherapy, or CDK4/6 inhibitors induce senCAFs in BC, leading to substantial secretion of immunosuppressive SASP factors that trigger both innate and adaptive immunosuppression. For innate immunosuppression, GM-CSF and CCL2 serve as chemotactic signals that recruit BM-derived MDSCs into the tumor microenvironment through the bloodstream. Under the influence of IL-6 and CCL2 within the microenvironment, MDSCs upregulate Arg-1 and iNOS, differentiating into a highly immunosuppressive phenotype. Concurrently, CCL2 and IL-6 induce TAMs to polarize toward an anti-inflammatory M2-like phenotype, further attenuating innate immune responses.For adaptive immunosuppression, senCAFs secrete CCL22 and CXCL12, recruiting Tregs into the tumor microenvironment; meanwhile, IL-10, TGF-β, and adenosine suppress DC antigen presentation by downregulating CD80/86 and MHC-II, thereby suppressing CD8^+^ T cell function and inducing CD8^+^ T cell exhaustion. Ultimately, these innate and adaptive immunosuppressive pathways converge to establish an immune evasion-promoting tumor microenvironment, which in turn drives enhanced SASP secretion by senCAFs—perpetuating a vicious cycle. CDK4/6, cyclin-dependent kinase 4/6; senCAFs, senescent cancer-associated fibroblasts; BC, breast cancer; SASP, senescence-associated secretory phenotype; GM-CSF, granulocyte-macrophage colony-stimulating factor; CCL2, C-C motif chemokine ligand 2; BM, bone marrow; MDSCs, myeloid-derived suppressor cells; IL-6, interleukin-6; TAM, tumor-associated macrophage; Arg-1, arginase 1; iNOS, inducible nitric oxide synthase; CCL22, C-C motif chemokine ligand 22; CXCL12, C-X-C motif chemokine ligand12; Tregs, regulatory T cells; IL-10, interleukin-10; TGF-β, transforming growth factor-β; DC, dendritic cell; CD80/86, CD80 and CD86 co-stimulatory molecules; MHC-II, major histocompatibility complex class II; CD8+T cells, CD8-positive T lymphocytes.

Although SASP is often discussed in the context of its tumor-promoting effects, this secretory phenotype originally emerged evolutionarily as a tumor-suppressive mechanism. This antitumor activity is mediated through a hierarchical cascade of SASP-driven immune surveillance (**Figure 2**). When cancer cells themselves undergo senescence, tumor proliferation is arrested, representing an intrinsic tumor-suppressive mechanism. In contrast, when senescence occurs in surrounding stromal cells, the release of SASP from these cells tends to promote tumor progression. An analysis based on approximately 2,000 BC samples revealed that senescence of cancer cells themselves may be more prevalent in BC than senescence of adjacent stromal cells. Specifically, patients with high overall expression levels of senescence-associated genes in BC tissues exhibited better prognoses, whereas those with low expression levels had worse outcomes (4). Notably, although the present study has identified the above correlations, the interpretation of these findings warrants caution due to inherent limitations in the study design, as this analysis could not distinguish whether senescence signals originated from tumor cells or stromal cells. In this regard, the study by Ye et al. demonstrated that senescent CAFs directly impair NK cell cytotoxicity, thereby compromising tumor immunosurveillance and promoting tumorigenesis. Selective elimination of senescent CAFs restores NK cell-mediated killing capacity, thereby reducing tumor incidence and recurrence (30), suggesting that the aforementioned methodological limitation may indeed reflect a genuine biological concern. Therefore, whether SASP exerts beneficial or deleterious effects in BC is fundamentally determined by the cellular identity of the SASP source, which constitutes a critical prerequisite for understanding its dual roles in this disease.

### 4.1 Immunosurveillance: recruitment of antitumor immune cells by SASP

Studies have demonstrated that CAFs, as the most abundant cell type in the TME, can directly impair natural killer (NK) cell cytotoxicity through extracellular matrix (ECM) production, whereas selective elimination of senescent CAFs reduces tumorigenesis in an NK cell-dependent manner (30). This finding indicates that the senescent status of distinct cell types within the TME may profoundly influence tumor progression by modulating immune cell function. Building upon this concept, the role of SASP in immunosurveillance has garnered increasing attention. In particular, the suppressive effect exerted by T-cell-mediated immune responses on tumor growth within the BC microenvironment provides compelling evidence supporting the notion that SASP influences BC progression through immune modulation.

Specifically, the small molecule NS1643 was employed to activate Kv11.1 potassium channels in BC cells, thereby inducing senescence in estrogen receptor-positive BC cells and subsequent SASP release. Further investigation revealed that this SASP effectively recruited CD4+ T cells and promoted their polarization toward a T helper 1 (Th1) phenotype, resulting in the secretion of the pro-inflammatory cytokine tumor necrosis factor-α (TNFα). Concurrently, senescent cancer cells upregulated their expression of TNFα receptor 1 (TNFR1), rendering them highly sensitive to TNFα-mediated killing and ultimately enabling immune-mediated elimination of senescent tumor cells (14).

In addition, neutrophils also participate in this process of immunosurveillance. Following the induction of senescence in BC cells, researchers observed marked upregulation of SASP components such as Interleukin-8 (IL-8) and serum amyloid A1 (SAA1), which recruited and activated neutrophils at the tumor site, contributing to the clearance of senescent cancer cells through phagocytosis. Notably, studies in specific BC models treated with palbociclib have confirmed that neutrophil recruitment and activation are intrinsic features of the senescent state itself, rather than being attributable to exogenous drug exposure per se; nevertheless, whether this effect persists under other chemotherapy-induced senescence contexts remains to be determined (7). Collectively, these findings elucidate the critical mechanisms by which SASP exerts precise antitumor immunosurveillance in BC through the recruitment and activation of distinct immune cell subsets.

### 4.2 Paracrine senescence: confining tumor expansion

Paracrine release of SASP-associated inflammatory factors mediates intercellular signaling within the senescent microenvironment and remodels the local tissue landscape. An early experiment utilizing primary human breast fibroblasts (BFs) demonstrated that senescent BFs secrete a key SASP component—soluble TGF-β—which subsequently transmits the senescent phenotype to adjacent neighboring cells, thereby exemplifying the characteristic paracrine transmission of senescence signals (12). Collectively, existing studies confirm that cellular senescence is transmissible via paracrine mechanisms; however, this transmission exhibits strong spatial restriction. These findings suggest that paracrine senescence transmission may erect a barrier that partially restricts local invasion and dissemination of cancer cells into adjacent normal tissue; however, SASP heterogeneity imposes spatial constraints on this propagation, limiting the uniformity of such protection.

### 4.3 Direct inhibition of tumor cell proliferation

As a hallmark feature of senescent cells, SASP directly contributes to tumor suppression through multiple mechanisms. *In vivo* experiments in mice have confirmed that senescence induction in BC cells by natural products inhibits the Janus Kinase (JAK2)/signal transducer and activator of transcription 3 (STAT3) signaling pathway, and the reduced activity of this pathway directly suppresses tumor cell proliferation, migration, and growth (34). This finding supports the role of SASP as a mediator of tumor suppression in specific contexts. Meanwhile, another study further elucidated the direct tumor-suppressive role of SASP from the perspective of the TME: Li M. et al. found that bone marrow-derived mesenchymal stem cells (BMSCs) activate the p21 signaling pathway through SASP secretion, and the subsequent expression of p21 inhibits the proliferation and survival of bone-metastatic BC cells (35). These results indicate that BC bone metastasis is constrained within a senescent microenvironment, with this inhibitory effect being achieved through the direct suppression of tumor cell proliferation by SASP.

## 5 The “blunt edge” of SASP: tumor-promoting effects in BC

### 5.1 Driving malignant progression of tumor cells: proliferation, stemness, and lineage conversion

A single-cell analysis revealed that a senescence-like tetraspanin-8 myofibroblastic CAF subset (TSPAN8 myCAFs) enhances the stemness of surrounding BC cells through SASP-derived Interleukin-6 (IL-6) and IL-8, thereby contributing to drug resistance in BC patients (6). Another study focusing on TNBC further demonstrated that although chemotherapy eliminates a fraction of cancer cells, the remaining treatment-insensitive cells are induced into a senescent state and subsequently release SASP. This SASP reshapes the TME in a paracrine manner and activates stemness-associated signaling pathways, thereby conferring cancer stem cell properties including self-renewal capacity, multipotent differentiation potential, and high tumorigenicity (36). Similarly, Lee CH et al. found that IL-6, as a component of SASP, suppresses MSN expression, leading to reduced STAT3 phosphorylation and thereby maintaining TNBC stemness (8). However, it should be noted that the magnitude of this stemness-enhancing effect varies across different BC subtypes. For instance, although CDK4/6 inhibitors can induce senescence and SASP production, the resulting SASP was found to activate neutrophils to enhance immunosurveillance rather than promote tumor stemness in HER2+ BC cells (7). Collectively, these findings indicate that SASP drives tumor-promoting effects by enhancing cancer stemness, although this effect is not universally applicable across all BC subtypes.

In addition, SASP exerts tumor-promoting effects by inducing aberrant proliferation of epithelial cells (37). Notably, SASP has also been implicated in lineage plasticity of BC cells. Beyond directly promoting tumor cell proliferation, SASP triggers neuroendocrine transdifferentiation in specific BC cell populations via the NF-κB/calcium signaling axis, leading to the emergence of a more aggressive and therapy-resistant cell population (38).

### 5.2 Remodeling the microenvironment to promote invasion and metastasis

SASP is closely associated with the molecular heterogeneity of invasive BC and often correlates with enhanced tumor aggressiveness (39). Furthermore, SASP-mediated reprogramming in senescent BC cells upregulates multiple effector molecules and reshapes the expression profiles of inflammation- and immune-related genes, offering new insights into BC pathobiology (40). Beyond its impact on molecular subtyping and gene expression, SASP-driven remodeling of the TME also extends to the direct modulation of cancer cell behavior. For instance, Wang et al. demonstrated that senescent fibroblasts secrete Granulocyte-Macrophage Colony-Stimulating Factor (GM-CSF) and basic fibroblast growth factor (bFGF), which activate the c-Jun N-terminal kinase (JNK) signaling pathway in BC cells, thereby inducing epithelial-mesenchymal transition (EMT). This process significantly enhances the migratory capacity of cancer cells and promotes distant metastasis (23). Collectively, these findings indicate that SASP contributes to tumor invasion and disease progression through multiple synergistic pathways.Similarly, SASP-mediated TME remodeling exerts tumor-promoting effects that vary across BC subtypes. A basic study in TNBC confirmed that chemotherapy-induced senescence elicits SASP secretion, and subsequent TME reprogramming by SASP promotes the development of chemoresistance in TNBC, thereby driving tumor progression (41). Furthermore, evidence from lung cancer models indicates that the combination of TGF-β signaling and hypoxia within the TME can induce deep senescence (D-senescence), resulting in the production of immunosuppressive SASP (42). Given that TGF-β signaling is frequently active in the TME of ER+ BC, and that regions of relative hypoxia commonly arise due to high proliferative activity and insufficient angiogenesis, it is plausible to hypothesize that TGF-β within the SASP may engage in cross-talk with ER signaling in this subtype. This notion is borne out in breast cancer as well. Reid et al. demonstrated that CAFs, via TGF-β signaling, suppress expression of ER-α, a critical oncogenic transcription factor in ER+ breast cancer, which thereby releases the brake on EMT and heightens the malignant phenotype; moreover, this suppression markedly diminishes tumor responsiveness to endocrine therapy, culminating in acquired resistance (13). In contrast, the interplay between SASP and EMT in HER2+ BC remains largely unexplored and warrants further investigation.

### 5.3 Immunosuppression: recruitment of immunosuppressive cells

SASP generated within the TME recruits immune cells such as myeloid-derived suppressor cells (MDSCs), induces them to acquire an immunosuppressive phenotype, and simultaneously suppresses the function of tumoricidal T cells and NK cells, thereby facilitating immune evasion in BC (19). This mechanism has been further corroborated in a clinical context. In a single-cell sequencing study of TNBC, comparison of tumor specimens from chemotherapy-sensitive and chemotherapy-resistant patients revealed a greater abundance of senescent tumor-associated macrophages (TAMs) in the resistant group, suggesting that chemotherapy-induced macrophage senescence promotes immunosuppression via SASP release and drives the development of drug resistance (43). This immunosuppressive effect has also been observed in other BC subtypes. In HER2+ BC, trastuzumab deruxtecan (T-DXd) can induce cellular senescence and SASP production, potentially establishing an immunosuppressive microenvironment (44).

### 5.4 Promoting angiogenesis

**Figure 3:**
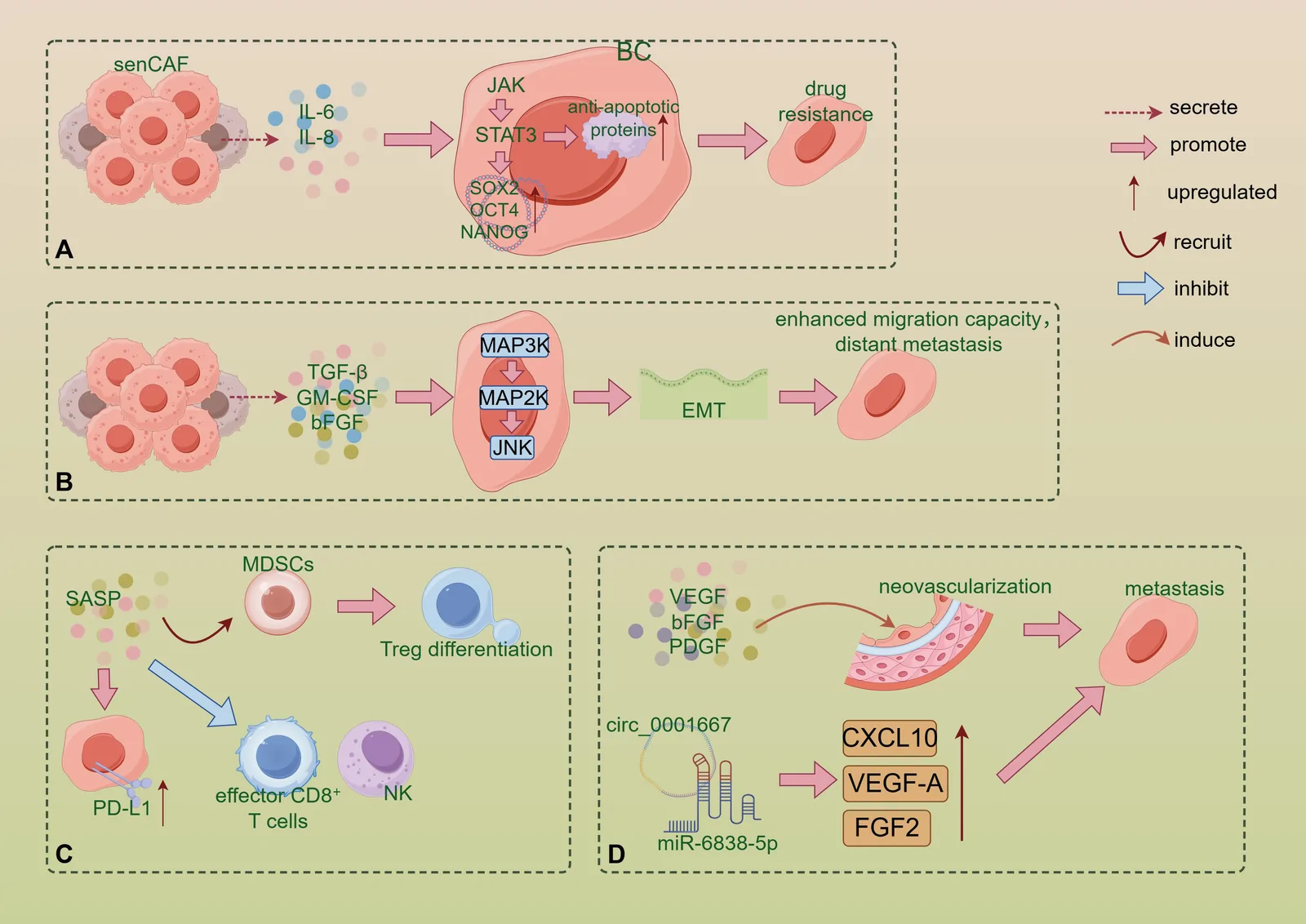
Schematic illustration of integrated pathways for SASP-driven BC progression. The SASP, secreted by senescent cells within the tumor microenvironment, drives BC progression through four principal pathways. **(A)** Cancer stemness and drug resistance: senCAFs release SASP components, among which IL-6 and IL-8 act on breast cancer cells in a paracrine fashion, activating the JAK/STAT3 signaling cascade and upregulating stemness transcription factors including SOX2, OCT4, and NANOG, thereby enhancing the self-renewal capacity of CSCs. Concurrently, STAT3 activation induces the expression of anti-apoptotic proteins, attenuating tumor cell sensitivity to chemotherapy and endocrine therapy and culminating in drug resistance. **(B)** EMT, invasion, and metastasis: TGF-β, a core SASP driver, cooperates with GM-CSF and bFGF to engage breast cancer cells, triggering the JNK signaling pathway and inducing EMT, which augments migratory and invasive capacities and enables distant metastasis. **(C)** Immune evasion: The SASP remodels the immune microenvironment by recruiting MDSCs and fostering Treg differentiation. Additionally, it upregulates PD-L1 on the surface of BC cells and suppresses the proliferation and cytotoxic function of effector CD8^+^ T cells and NK cells, thereby facilitating immune escape. **(D)** Angiogenesis: VEGF, bFGF, and PDGF within the SASP directly target vascular endothelial cells, eliciting tumor neovascularization. Furthermore, circ_0001667 derived from senescent endothelial cells competitively sponges miR-6838-5p, thereby elevating CXCL10 levels, which in turn stimulates VEGF and bFGF production, collectively fueling angiogenesis and metastasis in breast cancer. SASP, senescence-associated secretory phenotype; BC, breast cancer; senCAFs, senescent cancer-associated fibroblasts; IL-6, interleukin-6; IL-8, interleukin-8; JAK, Janus kinase; STAT3, signal transducer and activator of transcription 3; MAP3K, mitogen-activated protein kinase kinase kinase; MAP2K, mitogen-activated protein kinase kinase; JNK, c-Jun N-terminal kinase; SOX2, SRY-box transcription factor 2; OCT4, Octamer-binding transcription factor 4; NANOG, Nanog homeobox; CSCs, cancer stem cells; EMT, epithelial-mesenchymal transition; TGF-β, transforming growth factor-β; GM-CSF, granulocyte-macrophage colony-stimulating factor; bFGF, basic fibroblast growth factor; MDSCs, myeloid-derived suppressor cells; Treg, regulatory T cell; PD-L1, programmed death ligand 1; NK, natural killer; VEGF, vascular endothelial growth factor; PDGF, platelet-derived growth factor; CXCL10, C-X-C motif chemokine ligand 10; circ_0001667, circular RNA 0001667; miR-6838-5p, microRNA-6838-5p.

Within the BC microenvironment, not only cancer cells but also a substantial proportion of normal cells exist in a senescent state. These cells secrete SASP, releasing potent proangiogenic factors including vascular endothelial growth factor (VEGF) and platelet-derived growth factor (PDGF), which stimulate neoangiogenesis within the tumor, thereby creating favorable conditions for BC metastasis (19). In addition to SASP derived from these cellular sources, recent studies have uncovered novel proangiogenic mechanisms at the molecular level. In BC, circ_0001667 upregulates Chemokine (C-X-C motif) ligand 10 (CXCL10) expression by sponging miR-6838-5p, which in turn enhances the levels of VEGF-A and FGF2, promoting tumor angiogenesis and cell proliferation. Notably, as a key component of SASP, the elucidation of the regulatory mechanism underlying CXCL10 expression provides a molecular basis for understanding the tumor-promoting role of SASP in BC (18). Collectively, these tumor-promoting effects of SASP are subject to several limitations. Most studies have been conducted using *in vitro* co-culture experiments or immunodeficient mouse xenograft models, lacking parallel validation with human BC specimens. Consequently, definitive evidence confirming the clinical relevance of these phenomena in human BC remains insufficient. Furthermore, the tumor-suppressive and tumor-promoting effects of senescence are not inherently contradictory; rather, they represent different manifestations of the same biological process under distinct contextual conditions (**Figure 3**).

## 6 Targeting SASP: strategies to optimize BC therapy

Although substantial therapeutic advances have been achieved in BC treatment, SASP remains closely implicated in mechanisms of therapeutic resistance, and TIS has emerged as a significant challenge limiting treatment efficacy. Therefore, it is imperative to strategically harness the dual nature of SASP as a “double-edged sword”-amplifying its tumor-suppressive effects while concomitantly mitigating its tumor-promoting risks-thereby maximizing its therapeutic potential in BC. Currently, an increasing number of studies are exploring optimized combination therapeutic strategies targeting SASP, with the aim of achieving novel therapeutic breakthroughs in BC management.

### 6.1 Elimination of senescent cells

Targeting senescent cancer cells represents a critical yet challenging aspect of BC therapy. These cells evade therapeutic intervention through parallel immunosuppressive mechanisms, thereby rendering single-agent strategies ineffective. Therefore, a key therapeutic focus lies in the direct elimination of senescent cancer cells. Specifically, this requires a combined immunotherapeutic approach that concurrently blocks both the programmed death-ligand 1 (PD-L1) and Cluster of Differentiation 80 (CD80) immunosuppressive pathways, thereby effectively reversing tumor immune evasion and enabling the clearance of senescent cancer cells (45). Building upon this concept, the sequential therapeutic strategy of first inducing senescence and subsequently targeting senescent cells has shown considerable promise in BC.

Daglish et al. utilized TR-57, a caseinolytic protease P (ClpP) agonist, to successfully induce an irreversible senescent state in TNBC cells. During this process, the senescent cells released SASP factors and demonstrated markedly enhanced sensitivity to the B-cell lymphoma 2 (BCL-2) inhibitor venetoclax. The combined application of these two agents exerted a synergistic killing effect, leading to the efficient elimination of senescent tumor cells (46). In addition to this strategy, the combination of the poly-ADP-ribose polymerase (PARP) inhibitor talazoparib with radiotherapy has also been demonstrated to effectively induce senescence in BC cells. Subsequently, the addition of the BCL-2 inhibitor venetoclax enables the selective elimination of senescent cancer cells. This sequential therapeutic approach similarly exemplifies the “induce first, clear later” paradigm and exhibits a robust synergistic effect in enhancing antitumor efficacy (47). Collectively, these findings provide a highly promising sequential therapeutic strategy for TNBC, a particularly challenging subtype. By first inducing tumor cells into senescence followed by targeted elimination of senescent cells, this approach is expected to enhance antitumor efficacy while overcoming the drug resistance and relapse commonly associated with monotherapy, thereby holding significant promise for clinical translation.

In ER+ BC with low HER2 expression, CDK4/6 inhibitor-induced senescence has been shown to enhance the bystander effect of HER2-targeted antibody-drug conjugates (ADCs), providing a theoretical basis for the combination of senescence induction with ADC-mediated senescent cell clearance in this subtype (48). Furthermore, Vezzoli et al. reported that T-DXd-based ADC induces SASP production in HER2+ BC, thereby potentially increasing the risk of immune evasion (44). Therefore, combining T-DXd with senolytic agents to eliminate residual senescent cells may represent an effective strategy to counteract chemoresistance. Collectively, these studies demonstrate that combining existing therapeutic modalities with senescent cell clearance represents a broadly applicable strategy across BC subtypes. In future BC therapeutic research, the potential impact of SASP on treatment efficacy should be carefully considered.

It is noteworthy, however, that the beneficial effect of senescent cell clearance on reducing drug resistance may be achieved without additional adverse effects, provided that senescent cells are precisely eliminated without collateral damage to adjacent healthy cells. Achieving this level of precision represents a considerable technical challenge in the current landscape of BC therapy. Existing studies have documented several limitations of contemporary senescent cell clearance technologies, including inadvertent platelet toxicity, clearance of non-senescent cells, and failure to eliminate escaped senescent cells (49). Therefore, there is a pressing need to identify senescence-specific biomarkers with higher specificity to enable more accurate target identification. Concurrently, senolytic strategies should be further refined to achieve greater precision, employing the lowest effective dose within the minimal feasible target range, so as to protect neighboring normal cells to the greatest extent possible.

### 6.2 Modification of SASP

As mentioned above, SASP exhibits a dual nature. Therefore, while leveraging its anti-tumor effects, attention must be paid to its potential adverse effects, and appropriate intervention strategies should be implemented. For instance, melatonin supplementation or combined use of a cGAS inhibitor can effectively eliminate senescent macrophages, thereby restoring chemosensitivity (43).

A study using MCF-7 BC cells found that CDK4/6 inhibitor-induced senescent cancer cells exhibited significantly reduced SASP expression, indicating that CDK4/6 inhibitors exert anti-tumor effects by modulating SASP component expression (50). However, as this study was conducted in ER+ BC cells (MCF-7), whether CDK4/6 inhibitor-mediated SASP modulation has the same effect in other BC subtypes remains to be further validated. Related research has also been conducted in TNBC subtypes. A novel agent, Chalcone-9, has been found to possess significant anti-cancer activity, and its mechanism of action involves inhibiting the JAK-STAT signaling pathway, thereby downregulating the mRNA expression of SASP-related factors (51). TNBC subtypes are relatively insensitive to existing conventional targeted therapies, and TIS is a core mechanism of immune evasion in TNBC. Therefore, therapeutic strategies that target SASP expression may hold considerable promise in the treatment of TNBC. Future studies are needed to further explore whether inhibiting SASP activity in combination with immunotherapy can effectively suppress TNBC progression.IL-6 is one of the core components of SASP. The IL-6-JAK2-STAT3-calprotectin axis was first identified as a therapeutic target in HER2+ BC, and the study employed FDA-approved drugs, indicating strong clinical translational potential. Building on recent clinical research that applied this therapeutic strategy to TNBC, neoadjuvant chemotherapy combined with IL-6 pathway inhibition achieved a pathological complete response (pCR) rate of 62%, demonstrating that modulating IL-6 expression and activity is effective in BC treatment (52, 53).

## 7 Discussion

When interpreting the SASP-related literature in breast cancer, one critical aspect that cannot be overlooked is the classification of evidence levels. To critically appraise the cited studies, we categorized them into four evidence tiers in **Table 1**: level A comprises clinical or population-based evidence, including human specimen analyses and large cohort databases; level B comprises *in vivo* animal model evidence; level C comprises cell line or *in vitro* experimental evidence; and level D comprises review articles or conceptual frameworks. Among the various mechanisms discussed herein, only a fraction are currently supported by level A evidence. By contrast, the mechanistic elucidation of individual SASP components derives predominantly from cell lines or xenograft models, which still fall short of recapitulating the heterogeneity inherent to human breast cancer.

Certain seemingly contradictory findings can be reconciled when examined through the lens of evidence levels and tumor subtypes. For instance, Ungvari et al. reported that high expression of senescence-related genes predicts favorable prognosis in a pan-breast cancer cohort (4), whereas Park et al. demonstrated through subtype-stratified immunohistochemical analysis that SASP positivity is associated with poor prognosis in Luminal A tumors but favorable prognosis in triple-negative breast cancer (TNBC) (39). This apparent discrepancy may not reflect a genuine conflict in biological mechanisms but rather underscores the necessity of conducting stratified analyses by breast cancer subtype—a perspective emphasized throughout this review. Building upon the integrative framework proposed in **Figure 1**, future studies should systematically investigate the interplay among the three regulatory axes—cell of origin, temporal dynamics, and microenvironment—across different breast cancer subtypes. In particular, there is an urgent need for studies on therapy-induced SASP spanning all breast cancer subtypes to identify the time window in which the SASP switches from tumor-suppressive to tumor-promoting effects, thereby providing a rational basis for designing subtype-specific SASP-targeted intervention strategies.

In summary, accumulating evidence has established that SASP exerts a dual role in BC. On the one hand, it exerts anti-tumor effects through immune modulation and direct suppression of tumor progression. On the other hand, specific SASP components can promote BC development and progression by remodeling the TME and promoting tumor angiogenesis. Consequently, an increasing number of studies have begun to explore the therapeutic potential of targeting SASP in BC. Current research has developed various SASP-modulating therapeutic strategies, including the use of electrolyzed reduced water to induce sustained oxidative stress and promote BC cell senescence, the combination of plant-derived natural compounds with chemotherapy to induce senescence, and the inhibition of key molecules involved in cell growth to trigger senescence and SASP release in BC cells (50, 54, 55). These studies have focused on either the tumor-suppressive or tumor-promoting effects of SASP, aiming to suppress its pro-tumorigenic effects or enhance its anti-tumorigenic effects to achieve therapeutic goals.

However, the therapeutic application of SASP-targeting strategies in BC still requires further investigation. The dual role of SASP in BC arises from its complex composition of multiple secreted factors, whose expression levels and functional effects vary significantly due to high heterogeneity across different cellular contexts, temporal phases, and microenvironments (33). Based on this complexity, reprogramming the secretory profile of SASP may represent a promising therapeutic approach. For instance, rather than eliminating senescent cells entirely, selectively blocking the expression of pro-inflammatory, tumor-promoting components within SASP—focusing on attenuating their deleterious effects rather than inducing cell death—may be more suitable for treating BC, a highly heterogeneous solid tumor.

However, whether implementing senolytic or SASP-modulating strategies, accurate identification of senescent cells using advanced technologies remains a prerequisite. The greater the precision of senescence detection, the higher the therapeutic benefit and the lower the associated risks. Notable progress has been made in senescence detection methodologies; standardized gene signatures such as SenMayo have been established. Furthermore, a study based on 2,006 BC samples demonstrated that high expression of senescence-related genes is significantly associated with prolonged recurrence-free survival, highlighting the independent prognostic value of SASP signatures in BC (4, 56). Future research efforts dedicated to applying this analytical framework to transcriptomic datasets across various BC subtypes may facilitate the development of SASP-based biomarkers, enabling more precise identification of senescent cells. Concurrently, subtype-specific SASP characteristics could guide the implementation of targeted SASP therapies and accurate prognostic assessment.

In contrast to previous reviews that have primarily discussed tumor senescence from a pan-cancer perspective, this review elucidates the seemingly contradictory dual roles of SASP in tumor suppression and promotion across different BC subtypes at the mechanistic level, summarizes advances in senescence-targeted intervention strategies, and bridges fundamental mechanisms with clinical translational applications.
